# From safety science to operational reality: competing institutional logics in the translation of national patient safety recommendations

**DOI:** 10.1093/intqhc/mzag146

**Published:** 2026-09-21

**Authors:** Mohammad F Peerally, Elizabeth Sutton, Jane Ferguson, Kieran Walshe, Natalie Armstrong, Expert Advisory Group to REACTION, Sue Carr, Gillian Lewis, William Lea, Deinniol Owens, Claire Rudkin

**Affiliations:** Digestive Diseases Unit, Kettering General Hospital, United Kingdom; National Institute for Health and Care Research (NIHR) Greater Manchester Patient Safety Research Collaboration (GM PSRC), University of Manchester, Manchester, Oxford Road, Manchester, M13 9PL, United Kingdom; National Institute for Health and Care Research (NIHR) Greater Manchester Patient Safety Research Collaboration (GM PSRC), University of Manchester, Manchester, Oxford Road, Manchester, M13 9PL, United Kingdom; School of Medical Sciences, University of Leicester, United Kingdom; National Institute for Health and Care Research (NIHR) Greater Manchester Patient Safety Research Collaboration (GM PSRC), University of Manchester, Manchester, Oxford Road, Manchester, M13 9PL, United Kingdom; Health Services Management Centre, University of Birmingham, United Kingdom; Alliance Manchester Business School (AMBS), University of Manchester, United Kingdom; National Institute for Health and Care Research (NIHR) Greater Manchester Patient Safety Research Collaboration (GM PSRC), University of Manchester, Manchester, Oxford Road, Manchester, M13 9PL, United Kingdom; School of Health and Medical Sciences, City St George’s, University of London, United Kingdom

## Abstract

**Background:**

National patient safety investigation bodies conduct system-level investigations and produce recommendations intended to drive improvement. Evidence that such recommendations translate into meaningful improvement is weak, and the reasons for this are poorly understood. This paper examines how recommendations produced by the Health Services Safety Investigations Body (HSSIB), England’s independent national patient safety investigation body, are produced by investigators and received by the national organizations to whom they are directed.

**Methods:**

A qualitative multimethod design was used including documentary analysis of 22 investigation reports produced by the Healthcare Safety Investigation Branch (HSIB), predecessor to HSSIB, two focus groups with HSSIB patient safety investigators (*n* = 12) and semistructured interviews with 11 senior representatives of national organizations, recipients of HSSIB recommendations. Data were analyzed using reflexive thematic analysis, guided by institutional logics.

**Results:**

Documentary analysis showed that HSIB recommendations were nondirective, clustering at the weakest level of the hierarchy of risk controls and favouring administrative measures. Two competing institutional logics were constructed using reflexive thematic analysis. HSSIB investigators deploy a safety science logic focusing on systems thinking, collaborative engagement and nonpunitive learning, while recipient organizations enact a managerial-operational logic prioritizing resource management and operational feasibility. Four consequences follow: (i) recommendations are attenuated through nominal coproduction; (ii) recipients navigate priority thickets in which political salience, strategic fit, and resource availability govern implementation; (iii) accountability among recipients is diffuse; and (iv) HSSIB’s transparency carries limited traction within the managerial-operational logic.

**Conclusions:**

These conditions produce a paradox: HSSIB’s structural separation from the organizations it investigates, while central to its legitimacy as a learning body, can distance it from the operational realities that govern whether recommendations are acted upon. Overcoming this paradox requires deliberate logic-building strategies that address how recommendations are framed, how accountability is constructed, and how the governance landscape is coordinated.

## Introduction

System-level investigation of safety failures that transcend individual organizations is a long-established mechanism for learning in safety-critical industries such as aviation, rail and maritime. Such bodies issue nonpunitive system-wide recommendations and are independent of the organizations they examine [1]. In the past decade, England and Norway became the first countries to establish dedicated national patient safety investigation bodies on these principles [2, 3]. In England, the Healthcare Safety Investigation Branch (HSIB) was established in 2016 following recommendations from an Expert Advisory Group, an independent group chaired by the national director for patient safety and comprising patient representatives, senior clinicians, academics, air accident investigators and members of the Morecambe Bay investigation team [4]. HSIB became the Health Services Safety Investigations Body (HSSIB) in October 2023, operating as an independent public agency under the Health and Care Act 2022 [5], separate from the regulators, policymakers and provider organizations it investigates. This independence enables investigations to be carried out solely for learning, without attribution of blame or liability, distinguishing HSSIB from regulatory bodies [6, 7]. HSSIB’s recommendations are directed almost exclusively to national organizations rather than to individual NHS trusts, on the basis that system-level hazards require system-level correction.

However, concerns remain about whether safety investigations of any kind in healthcare translate into meaningful organizational change. Research into hospital-level incident investigations consistently shows that risk controls proposed in action plans lack strength and do not address identified contributory factors [8, 9]. Whether recommendations from national bodies such as HSSIB achieve greater impact than those generated by local, organization-level investigation**s** remains an open empirical question, crucial to healthcare systems with established national investigation bodies and those considering establishing them.

## Theoretical framework

Institutional logics theory offers a lens to study the translation of recommendations into organizational change. Institutional logics are the beliefs, practices, and cultural assumptions that determine how organizations understand problems, construct solutions, and determine what counts as legitimate action [10]. This perspective situates organizations within multiple societal orders, such as professions, corporate, and state [11].

Healthcare is characterized by institutional complexity being highly fragmented, populated by actors with distinct professional, managerial, and regulatory orientations, and subject to competing demands that no single logic can reconcile [12]. Thornton *et al.* [11] situate organizational-level logics within this broader inter-institutional system: the professions order values expertise and autonomy, while the corporate order is characterized by bureaucratic rules, organizational targets, and hierarchical accountability. These logics are not structurally deterministic, however: organizations facing the same competing demands respond variably, from resistance to selective adoption to hybridization, depending on organizational capacity for compromise [12]. When organizations guided by different logics interact, the result can be miscommunication, selective compliance, and institutional complexity [11]. The application of this framework to healthcare has shown how competing professional and managerial logics shape organizational responses to change, governance, and improvement initiatives [13, 14]. Whether multiple logics produce conflict depends on how compatible the prescriptions of each logic are, and how central each is to organizational functioning [15].

In this article, we treat competition between logics as something to be demonstrated empirically and its consequences as contingent, drawing on findings from a qualitative study of how recommendations from HSSIB are produced and received through the lens of institutional logics [11]. Following Bacchi’s ‘What’s the problem represented to be?’ approach, we treat HSSIB recommendations as constructions that reveal the assumptions and logic of their producers, while treating recipients’ interpretive responses as a separate, empirically observable site where those constructions are accepted, contested, or reworked [16].

## Materials and methods

### Study design

We used a qualitative multimethod design in three stages: documentary analysis of HSIB investigation reports; focus groups with HSSIB investigators; and semistructured interviews with senior representatives of recipient organizations. Two topic guides, developed through a rapid review of the literature on safety recommendations in healthcare, were used to structure the prompts of the focus groups and semistructured interviews. An advisory group comprising two senior clinicians, a patient safety specialist, a policy maker and a national investigator advised on design and findings. Ethical approval was granted by the University of Leicester Ethics Committee (43240-gs370).

### Documentary analysis

We analyzed 22 HSIB investigation reports, purposively sampled to span completed investigations across a range of clinical specialties (Supplementary material 1), published between 2021 and 2022. This window was selected at the design stage because these were the most recent investigations for which recipient organizations’ formal responses had been published, allowing time for responses to be lodged and acted on. We combined (i) categorization of recommendations using the Veterans Health Administration hierarchy of risk controls [17], which ranks interventions from weak administrative measures to stronger engineering and elimination controls (Fig. 1) by effectiveness and dependence on sustained human compliance [9, 18] and (ii) Bacchi’s ‘What’s the problem represented to be?’ approach [16]. The hierarchy provides a standardized and comparable measure of the strength of the ­recommendations proposed; Bacchi’s approach explores the assumptions that make the recommendations appear sufficient in addressing the underlying problems. Together they operationalize the material and symbolic dimensions of a recommendation.

**Figure 1:**
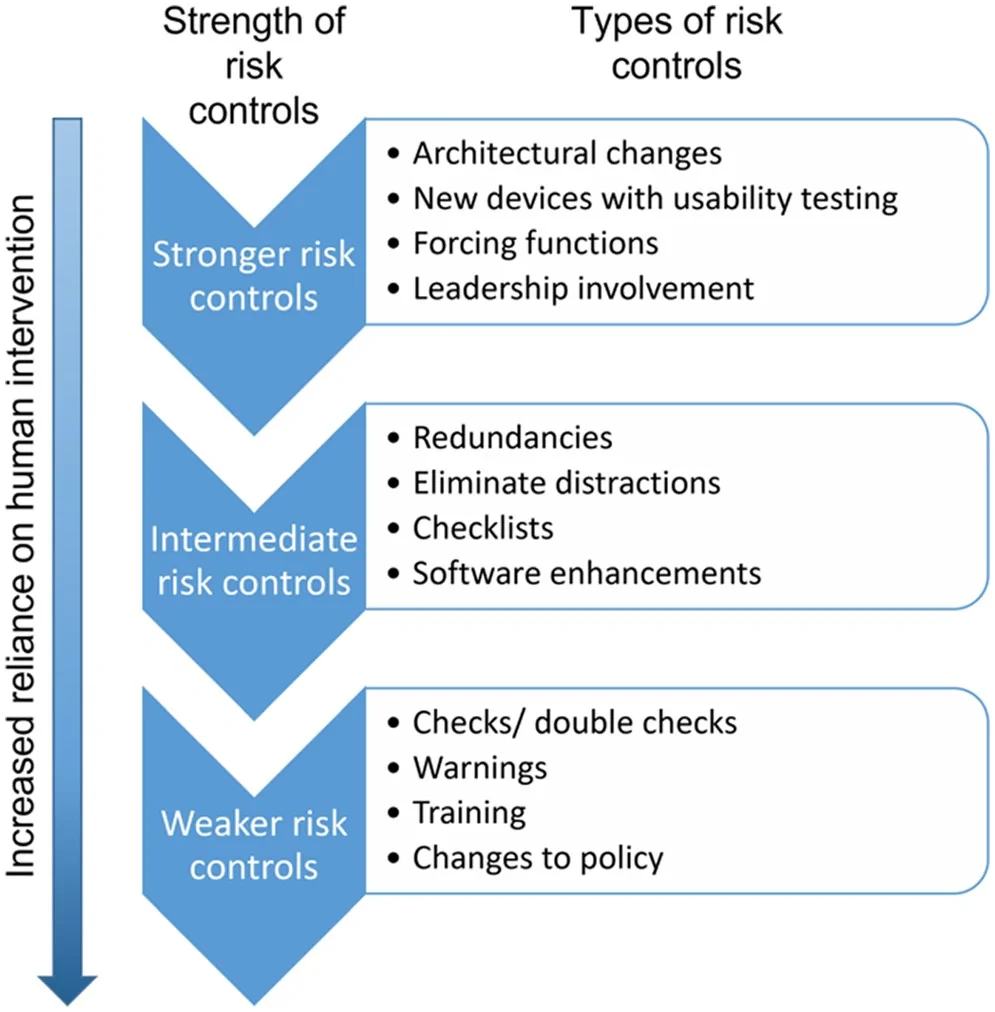
A diagrammatic representation of the hierarchy of actions, based on the Veterans Health Administration hierarchy (National Patient Safety Foundation, 2015).

### Focus groups

An experienced female qualitative researcher (ES) conducted two online focus groups via MS Teams®, with six HSSIB investigators in each (*n* = 12). Sessions lasted approximately 90 min and followed a semistructured topic guide (Supplementary material 2).

### Semistructured interviews

ES conducted 11 semistructured interviews, averaging 60 min, via MS Teams® with senior representatives of national organizations receiving HSSIB recommendations (Supplementary material 3), guided by a flexible topic guide (Supplementary material 4). Recipient organizations were identified from the documentary analysis as the most frequent addressees of HSIB recommendations, then sampled purposively to include system leadership (NHS England); regulation [Care Quality Commission (CQC), General Medical Council]; and standard-setting, professional and research bodies (National Institute for Health and Care Excellence, National Institute for Health and Care Research, and three medical royal colleges). Participants held senior policy, medical director, or quality and safety leadership roles and spoke in that organizational capacity rather than as frontline clinicians.

### Analysis of focus group and interview data

All focus groups and interviews were audio-recorded with written consent, transcribed verbatim by an approved third-party transcription service under a confidentiality agreement, then imported into NVivo 1.6.1. Reflexive thematic analysis was employed following Braun and Clarke’s approach [19]. LS inductively coded the data (Supplementary materials 5 and 6) and coproduced themes iteratively with MFP, then finalized these with all coauthors.

## Results

### Documentary analysis: the construction of safety recommendations

Recommendations in the 22 reports drew on safety science models such as AcciMap [20]. Recommendations included common terminologies which reflected collaboration such as ‘engage’ with partners, alongside nondirective and nonprescriptive phrasings such as ‘assess’ or ‘review and update’ existing guidance, and ‘consider’ further action. They rarely specified responsible individuals, resource requirements, or implementation mechanisms.

Of the 39 recommendations identified, 36 were categorized as weak (such as creation of new policies and training), three as intermediate and none as strong, based on the hierarchy of risk controls classification (Table 1). Read through Bacchi’s approach, this pattern represents the problem as one of insufficient awareness, guidance or coordination, for which the appropriate remedy is further deliberation by a national body.

**Table 1:** Report coding by recommendation type.

Report number and recommendations by type	Classification type		
	Weak	Intermediate	Strong
Report 1: flushing cannula	3	0	0
Report 2: emergency response to heart attack	3	0	0
Report 3: provision of mental health care to patients presenting at the emergency department	3	1	0
Report 4: wrong site surgery: wrong tooth extraction	3	0	0
Report 5: outpatient appointments intended but not booked	2	1	0
Report 6: management of chronic asthma in children aged 16 years and under	5	1	0
Report 7: wrong site surgery: wrong patient, invasive procedures in outpatient settings	1	0	0
Report 8: oxygen issues during the Covid-19 pandemic	5	0	0
Report 9: suitability of equipment and technology used for continuous fetal heart rate monitoring	3	0	0
Report 10: timely detection and treatment of cauda equina syndrome	5	0	0
Report 11: missed detection of lung cancer on chest X-rays of patients being seen in primary care	3	0	0
**Total**	**36**	**3**	**0**

National recommendations may appropriately target policy and training, which would then be *expected* to translate into stronger action locally. The qualitative data suggest this translation is far from straightforward.

Focus groups and interviews: competing institutional logics between national investigators and recipient organizations

### Investigators’ field-level safety science logic

HSSIB investigators draw on a field-level safety science logic which included a set of assumptions and practices shared across investigators from other high-reliability industries such as aviation, rail and maritime transport [7]. This logic was expressed through a systems-thinking orientation grounded in ‘work as done’ [21], a nonpunitive, learning-focused stance, and a facilitative rather than directive relationship with recipients.

*‘My approach is to understand how work is done in practice, what’s actually going on, and why that work cannot meet the expectations of a national body, standard, or policy.’* (FG1)

Without enforcement powers, investigators described a facilitative role, coordinating improvement through influence rather than mandate:

*‘More the conductor, helping to orchestrate…’* (FG1)

Coproduction of recommendations with recipients was framed by investigators as a deliberate strategy to increase implementability:

*‘We try to bring them on board… so that by the end, it doesn’t come as a surprise…’* (FG2)

### The managerial-operational logic of recipients of recommendations

Recipients’ responses, by contrast, were shaped by organizational-level logics: while a broadly shared managerial-operational orientation was evident across the recipient organizations, how it played out in practice depended on each organization’s own structures, priorities and internal cohesion. Recipients routed recommendations through different internal mechanisms: one recipient described prioritizing high-profile recommendations and putting them into a dedicated tracking system.

*‘We do internally at [organisation X] now have a recommendation tracker, and that is limited to… the very high-profile… recommendations.’* (INT03)

—while another prioritized them through council discussions:

*‘We are a membership body…. led by Council and they are all nominated clinicians, so they drive a lot of our business… quite often they take us in directions that probably [organisation Y] wouldn’t want to prioritise.’* (INT06)

Recipients spoke in a managerial language, using terms such as ‘business cases’, ‘cost-benefit analysis’ and ‘operational feasibility’. Investigators used the vocabulary of safety science: ‘hazard identification’, ‘hierarchy of controls’, ‘systems analysis’. Investigators read the divergence as a deficit in recipients’ understanding of the science of safety:

*‘… there’s a real lack of understanding (by recipients) of what risk really means….’* (FG2)

Recipients, however, experienced this divergence as inadequacy in how recommendations were framed and how they would be delivered. They described recommendations as lacking the evidential infrastructure necessary to mobilize organizational action:

*‘You haven’t demonstrated the business case for it. You haven’t effectively measured the scale of the problem that you’re trying to solve and assessed the scale of the cost.’ (INT 01)*

### The diffusion of accountability

Investigators described recipient organizations, particularly the large national bodies as internally fragmented. They were assemblages of departments that did not consistently communicate within their own boundaries:

*‘One organisation but many organisations within itself… directorates… may not speak to each other.’* (FG1)

Such segmentation created practical difficulties in identifying accountable individuals, particularly within organizations subject to frequent restructuring:

*‘I spend half my time trying to work out who to speak to in some of the big national bodies…’* (FG1)

This perceived fragmented structure of recipient organizations disrupted accountability. From the perspective of the safety science logic, a recommendation is addressed to a system; from the perspective of the managerial-operational logic, it must land with a named individual with the authority, resource, and mandate to act.

### Balancing coproduction of recommendations with independence of investigators

Investigators experienced independence as both a source of authority and a constraint on coproduction.

*‘… that tension between being independent and being collaborative… we have to balance that between also being able to challenge the healthcare system and sometimes make recommendations that stakeholders wouldn’t necessarily come up with themselves.’* (FG1)

Early engagement was their main mechanism for managing this tension:

*‘we try to bring them on board the investigation process as early as possible… so that hopefully… it doesn’t come as a surprise when we’re giving a certain recommendation…’* (FG2)

However, recipients’ accounts revealed that early engagement was unevenly experienced. Even when recipients were consulted, they reported limited meaningful engagement due to the maturity of the analytical process:

*‘It does feel sometimes that, particularly if we have something very significant to say in relation to a draft report, that it can feel a little bit late in the process.’* (INT 01)

Investigators also expressed frustrations with the coproduction of certain recommendations resulting in their ‘dumbing down’, where optimal recommendations, grounded in evidence from investigations were reframed to account for operational demands:

*‘The right thing… can’t actually be done because of a lack of finance or political will.’* (FG1)

*‘We’ve had to soften… because they’ve said, ‘that’s unachievable’.’* (FG2)

The result is a paradox embedded in the production process itself: coproduction mechanisms can increase likelihood of implementation, but carry an inherent risk that the analytical ambition of the safety science logic is progressively diluted in the pursuit of recipients’ acceptability.

### A crowded recommendation landscape

Both groups were frustrated by the accumulation of recommendations from multiple national bodies, which investigators recognized could render individual recommendations functionally invisible, while recipients felt overwhelmed with the volume:

*‘[the recommendations are] creating so much noise that people aren’t able to understand what they should be doing…’* (FG2)

*‘.there are so many organisations producing so many recommendations, we’re drowning in it.you’ve got to try and do something meaningful…I get written to by every organisation saying you must do something different…all they’re really doing…is saying here is another thing you’re liable for.’* (INT 07)

Attention was also unevenly distributed. Investigators observed that high-profile inquiries, particularly in maternity safety, displaced other priorities:

*‘Some of the maternity inquiries… have been given such high political importance… and they’re actually having to justify themselves against those particular recommendations…’* (FG2)

Here a state logic operates alongside the corporate one: recommendations carrying political salience were triaged upwards irrespective of the strength of the underlying safety analysis. For recipients, the result was ad hoc prioritization in which recommendations falling outside existing plans were deprioritized or disregarded (*‘ended up in the bin’*, INT07):

*‘it’s easier to [implement] certain recommendations if it’s already built in within our plan’* (INT 06)

#### Transparency as an accountability mechanism of limited currency

Unable to mandate their recommendations, investigators treated publication of their reports and of recipients’ formal responses as a substitute for authority:

*‘We publish the responses… our reports can be cited… raised… in an inquest.’* (FG1)

This reflects an accountability mechanism proper to the professions order, where the sanction for inaction is reputational rather than regulatory: publication creates pressure through visibility to peers and the public, in the absence of any power to compel.

But this is not the sanction to which recipient organizations answer. Operating within the corporate order, recipients are accountable through regulatory and hierarchical mechanisms involving enforcement action and performance ratings, against which a published response to recommendations carries little independent weight. Investigators seemed to sense this mismatch themselves, expressing scepticism about whether formal responses reliably indicated genuine intent and capacity to change practice rather than simply satisfying the requirement to respond:

*‘Anyone can write a response that… the minute you read it, you sort of think, OK, it may sound positive, but… they’re not going to do anything.’* (FG1)

## Discussion

### Statement of principal findings

This study explored the gap between patient safety recommendations produced by HSIB/HSSIB and the organizational change they are intended to trigger. We identified structural tensions between competing institutional logics to explain this gap. While investigators enact a safety science logic representing an empirical manifestation of Thornton *et al.*’s professions order [11], privileging evidence, systems thinking, collaborative engagement, and nonpunitive learning, recipients use a managerial-operational logic akin to Thornton *et al.*’s corporate order, prioritizing resource management and operational feasibility [11].

Four consequences follow. First, recommendations produced through safety science analysis risk being attenuated at the point of coproduction to maximize acceptability by recipients. Second, recommendations are triaged within ‘priority thickets’ [22], where the volume of competing recommendations renders rational prioritization problematic. Decisions around implementation of recommendations are governed by political salience, strategic fit, and resource availability of recipients.

Third, accountability is difficult to manage due to challenges in identifying named individuals with the mandate to act within recipient organizations. Fourth, transparency, HSSIB’s primary accountability lever, carries weight within the safety science logic but limited traction within the managerial-operational logic.

These conditions produce a paradox whereby the ‘independence’, characterized by its separation from the organizations investigated and protection from political interference, that legitimizes HSSIB’s recommendations inadvertently produces a structural separation from the operational realities governing their uptake.

### Strengths and limitations

The study’s multimethod design strengthens the credibility of our findings. We interpret our findings from the perspectives of both producers and recipients of recommendations. Use of institutional logics allows a theoretical explanation of the themes we identify. Limitations include analysis of only investigation reports published between 2021 and 2022 by HSIB, before it became HSSIB. We did not study the transfer of recommendations into improvements on the frontline of healthcare and focused only on HSSIB as a producer of recommendations. The study was conducted in England focussing on HSSIB and national bodies receiving its recommendations, but the mechanism and findings we describe are relevant to other analogous contexts where an independent nonregulatory investigative body directs recommendations to other organizations.

### Interpretation within the context of the wider literature

The dominance of recommendations, deemed weak on the hierarchy of risk controls [17, 18], reflects patterns evident at other levels of safety governance. Local investigations likewise target individual behaviour, training and the rewriting of policies [9, 23  24, 25], and investigators may attenuate recommendations in anticipation of resistance to resource-intensive proposals [24]. Recipients’ accounts of being overwhelmed match HSSIB’s own review [25] and the wider literature on recommendation volume [22]. Evidence from England’s Learning from Deaths programme points the same way, finding variable engagement, limited assessment of impact, and uncertainty about whether reported learning prevented further harm [26]. That the pattern recurs at national, local and programme level suggests a systemic property of healthcare safety governance rather than a defect of any single body.

Drawing on Bovens’ framework of public accountability [27], we identify an accountability vacuum: recommendations are formally directed to organizations as corporate actors, yet the scale and internal differentiation of those organizations means that no individual may hold the authority, capacity or capability to enact them. This is consistent with accounts of HSIB’s early years, in which concern was expressed that it risked being decoupled from the ‘engine that drives improvements’ and that only organizations ‘with teeth’ can make recommendations stick [28].

### Theoretical contribution

Healthcare applications of institutional logics have largely examined complexity within organizations, where hybrid professionals reconcile competing demands within their own practice [12, 14]. Our contribution is to locate the conflict at the boundary between the organization that produces a recommendation and the organization that must act on it. The ­recommendation is the artefact that carries a field-level logic across this organizational boundary: composed under the safety science logic, it must be actioned within the accountability and resourcing structures of the managerial-operational logic.

This boundary-crossing is negotiated, not resolved, at the point of coproduction. Investigators describe softening recommendations and diluting recommendations that cannot be enacted because of a lack of finance or political will. Coproduction is therefore not simply a mechanism for improving acceptability, as investigators frame it, but the site at which the safety science logic is translated into terms more recognizable within the managerial-operational logic.

Our findings also caution against reading logic conflict as inevitable. Prioritization of recommendations by recipient organizations was governed by political salience as well as resource constraints, suggesting a state logic operating alongside the corporate one. Where investigators and recipients had built durable working relationships, they navigated across logics without either being abandoned. Compatibility and centrality between logics, rather than the presence of two logics as such, appear to determine whether a recommendation is contested or absorbed [12]. We further propose strategies to bridge these logics in Table 2.

**Table 2:** Competing institutional logics in HSSIB recommendation implementation and bridging strategies.

Dimension	Safety science logic (HSSIB investigators)	Managerial-operational logic (recipient organizations)	Bridging strategy
Recommendation language and problem framing	HSSIB frames safety problems as systemic hazards requiring systemic correction. Recommendations employ nondirective, process-oriented language grounded in safety science methodology. Problems are represented as deficits in awareness, guidelines, or coordination.	Recipient organizations require clear, directive language specifying defined accountability mechanisms, costings, and implementation steps.	**What is already working:** HSSIB’s stakeholder consultation before publication of investigation reports and recommendations allows some refinement of wording. The ‘safety actions’ mechanism, where organizations take corrective steps during the investigation, demonstrates that early, plain-language dialogue can translate findings into operational change before formal publication. **Suggested innovation:** where possible, recommendations could be accompanied by a structured implementation note, codeveloped with recipients, that translates the safety science rationale into operational language, including the responsible party or parties within national bodies, indicative resource implications, and suggested implementation steps.
Professional identity and expertise	Investigators positioned themselves as possessing specialized expertise in safety science drawn from high-reliability industries. This professional identity was a source of institutional authority and legitimacy, and distinguished HSSIB from other bodies making recommendations.	Recipients did not share the vocabulary of safety science. Their institutional identity was grounded in governance, regulation, and operational management.	**What is already working:** investigators actively try to build relationships with recipient organizations across the investigation lifecycle. **Suggested innovation:** recipient organizations should invest in developing internal safety science literacy by training key personnel in human factors methodologies and systems thinking. HSSIB could support this through its ongoing educational and capability-building work.
Tension between independence and collaboration	Investigators valued their independence while simultaneously producing recommendations collaboratively with recipients.	Recipients valued early involvement but reported that consultation frequently occurred late. Engagement was experienced as procedural rather than substantive.	**What is already working:** early stakeholder engagement is already embedded in HSSIB’s investigative model and is consistently identified by both parties as a mechanism to improve uptake of recommendations. The safety action mechanism demonstrates what genuine early coproduction can achieve. **Suggested innovation:** a structured two-stage process: Stage 1 preserves full investigative independence for evidence gathering and analysis; Stage 2 creates a dedicated feasibility engagement phase in which recipients can provide input on recommendations and their implementation without altering the safety science conclusions.
Transparency as an accountability mechanism	HSSIB maintains its reputation through the transparency of its outputs. Investigators acknowledged having ‘no teeth’ to enforce recommendations but argued that transparency and their reputation provided meaningful leverage.	Recipients experienced published responses as institutional liability rather than learning.	**What is already working:** publication of investigation reports, recommendations, safety actions and recipients’ responses creates a signal of reputational accountability. **Suggested innovation:** published responses could be followed up after a defined interval, for example a 12-month follow-up submission by recipient organizations.
Priority thickets and diffusion of accountability	HSSIB investigators were aware of the crowded recommendation landscape but operated from a safety science obligation: identifying a hazard creates a duty to produce a recommendation. The volume of recommendations from multiple bodies was understood as a systemic problem, but not as a reason to withhold a recommendation arising from robust investigation.	Recipients described ‘drowning in recommendations’ from multiple national bodies, leading to ad hoc triage. Implementation was dependent on whether a recommendation aligned with existing organizational priorities. Accountability for implementation was diffuse.	**What is already working:** some recipient organizations have developed internal processes for triaging recommendations. **Suggested innovation:** a body operating at a national level, such as the National Quality Board or Department of Health and Social Care in England, should curate, deduplicate and sequence recommendations across bodies, providing prioritization signals. Each recommendation should identify a senior responsible owner within the recipient organization, supported by a named delivery team with clear authority, resources, timelines, and review mechanisms.

### Implications for policy, practice, and research

The proposed absorption of HSSIB into the CQC [29] risks reproducing this conflict in a different setting. The CQC holds organizations to account through inspection, rating and enforcement; placing HSSIB within that structure, without ring-fencing its investigative independence and safety science identity, would set its logic against a regulatory-compliance one.

Addressing priority thickets requires coordination. A recommendations repository, hosted within the National Quality Board or Department of Health and Social Care [23], could curate, deduplicate and sequence recommendations across bodies.

Each recommendation should identify a named senior ­responsible owner, supported by a named delivery team with clear authority, resources, timelines and review mechanisms; accountability resting on an individual alone is fragile, whereas team-level accountability is sustained relationally [30]. HSSIB should produce implementation summaries to accompany recommendations, setting out accountable functions and indicative resource implications, including the economic appraisal whose absence recipients identified as a barrier. Recipient organizations would in turn benefit from investment in safety science literacy, which national investigators such as HSSIB are well placed to support through investigator training and capability-building work.

Because HSSIB directs recommendations almost exclusively to national bodies, provider organizations receive them only indirectly. Directing some stronger and more operationally specific recommendations to NHS trusts where safety problems are most prominent would allow framing that resonates with local operational realities.

Future research should examine the translation of national recommendations into frontline outcomes, test whether these dynamics operate in other healthcare systems, and evaluate logic-bridging strategies.

## Conclusions

The gap between national patient safety investigation and meaningful organizational change can be explained by the divergence between the safety science logic of HSSIB investigators and the managerial-operational logic of recipients. In conflict, these logics yield weaker recommendations, compounded by an accountability vacuum and by the volume of recommendations recipient organizations must absorb. Resolving these tensions requires deliberate logic-bridging strategies addressing how recommendations are framed, how accountability for acting on them is structured, and how a fragmented governance landscape is coordinated. Without such structural attention, the aspiration that independent national investigation drives meaningful patient safety improvement will remain, paradoxically, unrealized.

## Supplementary Material

mzag146_Supplementary_Data

## Data Availability

The data that support the findings of this study are not publicly available due to ethical restrictions on participant confidentiality. Requests for access to anonymized data extracts may be directed to the corresponding author.
