# Supplementary material for: From safety science to operational reality: competing institutional logics in the translation of national patient safety recommendations
Source: Int J Qual Health Care. 2026 Sep 21;38(4):mzag146. doi: 10.1093/intqhc/mzag146 (PMC13690090; doi:10.1093/intqhc/mzag146)
Supplement: Supplementary file 1 [file mzag146_supplementary_data.zip › Supp material 1 table of reports.docx]

**22 reports**

**2021-22**

**Area of care**: Secondary impatient (14), Ambulance or 999 (3), Other (3 – MH and emergency care; wrong site; children and gender dysphoria services); Primary or community (1); out of hours GP or 111 (1)

**Recipients**: Royal College of Anaesthetics; Centre for Perioperative Care; NHS England and NHS Improvement; Association of Ambulance Chief Executives; NICE; Royal College of Emergency Medicine; Royal College of Psychiatrists; CQC; NHSX; PHE; British Association of Spine Surgeons; Royal College of Radiologists; Chair of the NHS System-wide Paediatric Observations Tracking (SPOT) Programme; Association of Ambulance Chief Executives; College of Paramedics; Royal College of Paediatrics and Child Health; NIHR; Medicines and Healthcare products Regulatory Agency; NHS Digital; DHSC; NHS Resolution; Resuscitation Council (UK); Health Education England; NHS England.

| Residual drugs in intravenous cannulae and extension lines | 2021 | Secondary Inpatient | HSIB recommends that the **Royal College of Anaesthetists and Centre for Perioperative Care** work with relevant stakeholders - such as the Association of Anaesthetists, College of Operating Department Practitioners, and Association for Perioperative Practice - to review, update and integrate new guidance on the surgical safety checklist 'Sign Out' process. Specifically, the guidance should be updated in relation to the flushing of cannulae and extension lines by strengthening the current administrative barriers, considering the hierarchy of hazard control, and the issues identified by the HSIB investigation.    HSIB recommends that the **Royal College of Anaesthetists** reviews its 'Guidelines for the provision of anaesthetic services' regarding the planning and oversight of perianaesthetic care in non-theatre settings.    This should include:   - guidance to assist anaesthetic departments to consistently plan for short-notice or emergency cases which take place in the non-theatre setting - planning which considers and mitigates against unexpected changes in conditions. |
| --- | --- | --- | --- |
| Emergency response to heart attack | 2021 | Ambulance or 999 | HSIB recommends that **NHS England and NHS Improvement** revise the Ambulance Clinical Quality Indicator: Clinical Outcomes for ST-elevation myocardial infarction to reflect each element of the call to balloon response and review this indicator alongside the critical time standards workstream.    HSIB recommends that **NHS England and NHS Improvement** support the Joint Ambulance Improvement Programme to respond to emerging risks and research highlighting factors impacting on effective ambulance response.    HSIB recommends that the **Association of Ambulance Chief Executives**, working with the College of Paramedics and cardiology specialists, produces a position statement on the use of pre-hospital thrombolysis by paramedics. |
| Provision of mental health care to patients presenting at the emergency department | 2021 | Other | It is recommended that **NHS England** ensures there is a sustainable funding model to support 24/7 urgent and emergency mental health liaison services in acute general hospitals with emergency departments.    It is recommended that the **National Institute for Health and Care Excellence** review and amend guidance for the management of self- harm in the emergency department.    It is recommended that the **Royal College of Emergency Medicine**, in conjunction with the **Royal College of Psychiatrists**, develops and disseminates national guidance for emergency department practitioners to standardise the initial assessment of a person presenting following a mental health emergency.    It is recommended that the **Care Quality Commission** reviews and updates its inspections criteria for emergency departments to ensure equal weight is given to the quality of care provided to people with urgent mental health problems as they do to people with urgent physical health. This would be consistent with its commitment to parity of esteem for mental health. |
| Wrong site surgery – wrong tooth extraction | 2021 | Other | HSIB recommends that **NHS England and NHS Improvement** should review the Never Events policy and framework and include content to explicitly define the criteria that need to be satisfied for any control to be considered a ‘strong systemic protective barrier’ |
| Outpatient appointments intended but not booked after inpatient stays | 2021 | Secondary Outpatient | HSIB recommends that **NHS England and NHS Improvement** develops standards and an operating framework that describes the assurance required for all outpatient appointment booking processes, including after an inpatient stay. The assurance should include feedback mechanisms which provide safeguards that intended outpatient appointments are booked. Ideally, solutions will use technology and automation to create resilience and efficiency so that there is less reliance on staff vigilance.    HSIB recommends that **NHSX**’s What Good Looks Like programme includes a requirement for organisations to be responsive to HSIB reports and recommendations within the ‘Safe Practice’ section of its guidance. |
| Management of chronic asthma in children aged 16 years and under | 2021 | Secondary Outpatient | HSIB recommends that **NHS England and NHS Improvement**, as a commissioning body, supports local systems to implement evidence-based interventions, such as standardised information and wheeze management plans, for the parents/carers of pre-school children. This will be undertaken in conjunction with the British Paediatric Respiratory Society.    HSIB recommends that **NHS England and NHS Improvement** reviews the recommendations arising from the National Review of Asthma Deaths to prioritise and ensure the implementation of recommendations that are outstanding.    HSIB recommends that **NHS England and NHS Improvement** supports clinical experts to work with professional bodies to develop training competencies for healthcare professionals with responsibility for caring for children with suspected or confirmed asthma.    HSIB recommends that **NHS England and NHS Improvement** and **NHSX** identify and integrate data items into information technology systems to develop a greater understanding of the risk factors present in the community.    HSIB recommends that **NHSX**, supported by NHS England and NHS Improvement, implements a discovery programme into the roadmap for the digital personal child health record focused on developing support, self-reporting and alerting for asthma self-care.    HSIB recommends that **Public Health England** develops resources for young people and their parents/carers to raise awareness and enable them to self-manage asthma more effectively. |
| Wrong site surgery – wrong patient: invasive procedures in outpatient settings | 2021 | Secondary Outpatient | HSIB recommends that **NHS England and NHS Improvement** leads a review of risks relating to patient identification in outpatient settings, working with partners to engage clinical and human factors expertise.  This should assess the feasibility to enhance or implement layers of systemic controls to manage these risks. It should also consider existing challenges relating to the usability and practice of including the NHS unique identifier in patient identification processes, and consider technological solutions to support its use. |
| Oxygen issues during the Covid-19 pandemic | 2021 | Secondary Inpatient | HSIB recommends that **NHS England and NHS Improvement** urgently issue definitive guidance on the role, function, and key attendees of the medical gas committee. This guidance should identify and encourage key multidisciplinary relationships and board level reporting of medical gas issues.    HSIB recommends that **NHS England and NHS Improvement** review and further specify the key roles, responsibilities and competencies of individuals identified in the health technical memorandum (HTM) for medical gas pipeline systems, including identifying how the appointment and training of designated officers may be supported.    HSIB recommends that **NHS England and NHS Improvement** implement a process to provide ongoing assurance on the qualifications and experience of individuals identified in the health technical memorandum (HTM) for medical gas pipeline systems (MGPS), including how MGPS Authorising Engineers, or their subcontractors, are appointed by NHS trusts.    HSIB recommends that **NHS England and NHS Improvement** completes ongoing work to review, revise and reissue the health technical memorandum (HTM) for medical gas pipeline systems (MGPS). An updated HTM should reinforce multidisciplinary team working and include:     - Updated advice on the type and design of MGPS infrastructure recommended for NHS trusts. - Enhanced processes to encourage shared working between clinical and nonclinical teams on MGPS issues. - Specifications for the relevant levels of competence and training for NHS staff on MGPS. - Any updated processes or guidance generated in response to the other safety recommendations specified in this report (R/2021/120, R/2021/132, R/2021/133).     HSIB recommends that the **Care Quality Commission** reviews and adapts its assessment model for NHS hospital estates to ensure greater scrutiny of estates related safety concerns. |
| Suitability of equipment and technology used for continuous fetal heart rate monitoring | 2021 | Secondary Inpatient | HSIB recommends that **NHS England and NHS Improvement** amends the ‘Saving Babies’ Lives care bundle version 2’ to enhance the role of the ‘fetal monitoring lead’ to include, training and competency checks of all maternity staff on the use and functionality of cardiotocograph (CTG) equipment.    HSIB recommends that **NHS England and NHS Improvement** amends the ‘Saving Babies’ Lives care bundle version 2’ to remove specific references to Dawes-Redman and instead use a generic term such as ‘computerised cardiotocograph (CTG) analysis’.    HSIB recommends that the **National Institute for Health and Care Excellence** considers reviewing its telemetry recommendation as part of the current update of clinical guideline CG190, taking into account the existing evidence and the findings of this report. |
| Timely detection and treatment of cauda equina syndrome | 2021 | Secondary Inpatient | HSIB recommends that the **British Association of Spine Surgeons**, supported by the Royal College of Surgeons of England and the Royal College of Emergency Medicine, develops a decision-making tool to support the identification of patients who need an immediate MRI for suspected cauda equina syndrome (which may result in the patient being transferred for MRI if this is not immediately available at the assessing site).    HSIB recommends that the **British Association of Spine Surgeons** oversees the development of national guidance to identify how ‘urgent’ and ‘emergency’ requests for scans for suspected cauda equina syndrome are defined and prioritised.    HSIB recommends that guidance is developed by the **Royal College of Radiologists**, supported by the Society and College of Radiographers, stating that all hospitals should reserve the first MRI slot of the day for patients with suspected cauda equina syndrome who do not meet the criteria for an ‘emergency’/immediate scan overnight.    HSIB recommends that the **National Institute for Health and Care Excellence** updates its current low back pain guideline to include the symptoms and initial management of cauda equina syndrome. This update should include a review of the role of supplementary investigations, such as bladder scanning, in patients with suspected cauda equina syndrome.    HSIB recommends that **NHS England and NHS Improvement** develops a national cauda equina syndrome pathway. This should define the safety-critical elements of the pathway and highlight areas that can be adapted locally. |
| Missed detection of lung cancer on chest X-rays of patients being seen in primary care | 2021 | Primary or Community | HSIB recommends that the **National Institute for Health and Care Excellence** reviews its current safety netting advice to healthcare professionals with respect to the investigation of possible lung cancer. The wording of the advice should be amended as required to make it clearer what should be offered to patients with ongoing, unexplained symptoms who have had a negative chest X-ray.    HSIB recommends that **NHSX**, in collaboration with relevant stakeholders such as The Royal College of Radiologists and The Society and College of Radiographers, develops guidance to support independent benchmarking and validation of artificial intelligence algorithms for the identification of lung diseases such as cancer.    HSIB recommends that the **National Institute for Health Research** assess the priority, feasibility and impact of future research to address whether low-dose computed tomography (CT) is clinically and cost-effective for the diagnosis of lung cancer in symptomatic patients seen in primary care and consider the most appropriate way of building up the evidence base on this topic. |
| Surgical care of NHS patients in independent hospitals | 2021 | Secondary Inpatient | HSIB recommends that **NHS England and NHS Improvement** ensures that effective processes have been implemented in integrated care systems to identify local capability and capacity of their independent acute hospitals.    HSIB recommends that **NHSX** expands its work programme addressing the challenges associated with interoperability of information systems used in healthcare to include transfer of information between the NHS and independent sector in support of safe care delivery.    HSIB recommends that the **Care Quality Commission** reviews and appropriately develops its methodology for regulatory assurance of arrangements between NHS and independent providers for the provision of care across care pathways. This is to include any screening and risk management processes used to ensure the safe transfer of care between providers.    HSIB recommends that the **Care Quality Commission** incorporates regulatory assurance of surgical pathways between providers at a system level when developing its methodology for the regulation of integrated care systems.    HSIB recommends that **NHS England and NHS Improvement** reviews models of perioperative care for their value and impact. This should inform future work to support implementation of a standardised approach, based on evidence, across all healthcare providers that deliver surgical services.    HSIB recommends that **NHS England and NHS Improvement** establishes a process to ensure that findings of the National Institute for Health Research’s policy research programme into frailty in younger patient groups are reviewed and acted upon. |
| Recognition of the acutely ill infant | 2021 | Ambulance or 999 | HSIB recommends that the **Chair of the NHS System-wide Paediatric Observations Tracking (SPOT) Programme** ensures that the Association of Ambulance Chief Executives, community NHS 111 providers and primary care services are integral members of the NHS SPOT Programme.    HSIB recommends that the **Chair of the NHS System-wide Paediatric Observations Tracking (SPOT) Programme** ensures that any resources produced include examples of children and young people with non-white skin showing signs of serious illness.    HSIB recommends that **NHSX** develops national standards describing the electronic deployment of the NHS System-wide Paediatric Observations Tracking (SPOT) e-PEWS (the digital version of the Paediatric Early Warning Score tool), in collaboration with the NHS England and NHS Improvement SPOT Programme. This should include specifications for data capture, calculation of the score and escalation status, and also the display of the information and connectivity with other digital systems.    HSIB recommends that the **Association of Ambulance Chief Executives** works together with the ambulance services to share best practice in relation to paediatric training, education resources, frequency and types of training, and that it collates and shares areas of best practice.    HSIB recommends that the **College of Paramedics** works with partners and higher education providers to develop, agree and implement standards for paediatric education for the future ambulance service workforce. |
| Weight-based medication errors in children | 2022 | Secondary Inpatient | HSIB recommends that the **Royal College of Paediatrics and Child Health** identifies the best practice principles for effective paediatric ward rounds in relation to medicines, and disseminates them to its members.    HSIB recommends that the **National Institute for Health Research** assesses the priority, feasibility and impact of future research on processes for second checking medication, and considers the most appropriate way of building up the evidence base on this topic.    HSIB recommends that the **Medicines and Healthcare products Regulatory Agency** works with the manufacturers of electronic prescribing and medicines administration systems to provide guidance on their obligations under the Medical Devices Regulations 2002 (as amended).    HSIB recommends that **NHS Digital and NHSX** promote the organisational requirements for digital clinical safety, including organisations’ responsibilities in terms of safety cases and clinical safety officers, to encompass system functionality and processes.    HSIB recommends that the **Care Quality Commission** (CQC) reviews whether a provider’s assurance of its compliance with the Clinical Risk Management standard specific to electronic prescribing and medicines administration systems in healthcare, can form part of the CQC’s developing regulatory model. |
| Maternity pre-arrival instructions by 999 call handlers | 2022 | Ambulance or 999 | HSIB recommends that **the Department of Health and Social Care** commissions the National Institute for Health and Care Excellence to work with relevant stakeholders to develop guidance for maternity emergencies in the non-visual, non-clinician-attended environment.    HSIB recommends that the **Department of Health and Social Care** identifies a suitable regulatory mechanism to provide formal oversight of 999 maternity pre-arrival instructions across NHS-funded care in England.    HSIB recommends that **NHS England and NHS Improvement** develops the content of the patient safety incident investigation (PSII) standards to further support cross-boundary investigations. |
| Emergency neonatal blood transfusion at birth following acute blood loss during labour and/or delivery | 2022 | Secondary Inpatient | HSIB recommends that **NHS Resolution**, working with relevant specialities through the clinical advisory group, amends the maternity incentive scheme guidance for year five to include the neonatal team as one of the professions required to attend multi-professional training.    HSIB recommends that the **Resuscitation Council (UK)**’s Newborn Life Support training course highlights that neonatal resuscitation teams should consider fetal blood loss in the event of neonatal resuscitation that includes chest compressions. In addition, this consideration should be included in the guidance to support the newborn life support algorithm. |
| Clinical decision making: diagnosis of pulmonary embolism in emergency departments | 2022 | Secondary Inpatient | HSIB recommends that **Health Education England** works with appropriate professional bodies to develop and implement a strategy for supporting the education and training of clinical practitioners that can facilitate the development of decision-making skills. This strategy should consider the use of innovative approaches such as simulation and immersive learning.    HSIB recommends that the **National Institute for Health and Care Excellence** reviews the findings of this investigation in relation to its guidance NG158, ‘Venous thromboembolic diseases: diagnosis, management and thrombophilia testing’, and updates the guidance if required.    HSIB recommends that the **Royal College of Emergency Medicine** promotes best practice around diagnostic decision making with respect to patients with potential symptoms and signs of pulmonary embolism. |
| Provision of care for children and young people when accessing specialist gender dysphoria services | 2022 | Other | HSIB recommends that **NHS England and NHS Improvement** incorporates the findings of this investigation into plans to further review and develop the service specifications for specialised gender dysphoria services. This should include further work with relevant stakeholders to:     - Identify the role of relevant voluntary and charitable sector organisations in supporting patients with gender identity concerns and facilitate information sharing between these organisations and regional professional support services.      - Identify work to improve the transfer of care, management, and proactive risk assessment of patients who are moving from the Gender Identity Development Service waiting list to a gender dysphoria clinic waiting list. |
| Decontamination of surgical instruments | 2022 | Secondary Inpatient | HSIB recommends that the **Care Quality Commission** reviews and ensures that the approach used by healthcare providers to assure themselves that in-house or externally contracted sterile services for decontamination are safe and can identify and respond to patient safety concerns.    HSIB recommends that the **Department of Health and Social Care** assesses the benefits of a single regulatory and assurance framework for sterile services departments and implements the findings as required.    HSIB recommends that **NHS England and NHS Improvement** amends Health Technical Memorandum 01-01 to define ‘top management’ and its commitment to quality, and that external independent audits are reported directly to the responsible executive director in a trust who is accountable for the service, not just the certified department.    HSIB recommends that **NHS England and NHS Improvement** develops a competency framework, stating skills, qualifications and professional registration as required, for all sterile services staff and includes it in Health Technical Memorandum 01-01. |
| Medicine omissions in learning disability secure units | 2022 | Secondary Inpatient | HSIB recommends that **NHS England and NHS Improvement** reviews and updates all health building guidance relating to learning disability secure units to reflect current clinical guidance on ensuring the design and layout provides a suitable environment for patients and staff.    HSIB recommends that **NHS England and NHS Improvement** develops the ongoing work to improve the retention of learning disability nurses, in line with the intent of the All-England plan for learning disability nursing. |
| The use of an appropriate flush fluid with arterial lines | 2022 | Secondary Inpatient | HSIB recommends that the **Medicines and Healthcare products Regulatory Agency** engages with other national regulators and relevant stakeholders to develop design guidance on labelling and packaging specific to fluids to reduce selection errors.    HSIB recommends that the **Medicines and Healthcare products Regulatory Agency** reviews and acts on the available evidence to regulate for the use of pressure infusion bags that allow fluid labels to be read when inflated.    HSIB recommends that the **Medicines and Healthcare products Regulatory Agency** communicates to all relevant stakeholders and acts on the available evidence concerning the management of the risks associated with arterial transducer line sets.    HSIB recommends that the **Department of Health and Social Care**, once post-market surveillance data is available, involves relevant stakeholders including the Association of Anaesthetists’ review and determine appropriate actions that could be taken to further mitigate the risk of blood sample contamination by the flush fluid when using arterial transducer line systems.    HSIB recommends that the **Association of Anaesthetists** works with relevant professional organisations to revise existing national guidance to manage the risks of contamination by the flush fluid when using an arterial line to take a blood sample.    HSIB recommends that the **Care Quality Commission** reviews the recommendations from the Association of Anaesthetists on how to manage the risks of contamination by the flush fluid when using an arterial transducer line and determines any appropriate actions for the oversight of governance and assurance arrangements within NHS providers following. |
| NHS 111’s response to callers with Covid-19-related symptoms during the pandemic | 2022 | Out of hours GP or 111 | HSIB recommends that **NHS England** ensures any Single Service contract or additional services contracts reflects the minimum requirements of the core NHS 111 service for audio-recording calls.    HSIB recommends that **NHS England** reviews the risks associated with increased use of telephone triage in response to national healthcare emergencies. Consideration should be given to applying any recommendations of this review across telephone triage services within the wider healthcare setting. |
