# Supplementary material for: From safety science to operational reality: competing institutional logics in the translation of national patient safety recommendations
Source: Int J Qual Health Care. 2026 Sep 21;38(4):mzag146. doi: 10.1093/intqhc/mzag146 (PMC13690090; doi:10.1093/intqhc/mzag146)
Supplement: Supplementary file 1 [file mzag146_supplementary_data.zip › Supplementary material 2- Topic Guide focus groups revised 29.5.2024.docx]

**From Recommendation to Action (ReACTION): Exploring the Translation of National Guidance into Organisational Change for Patient Safety**

**Focus Group Topic Guide**

***Before starting:***

***Ensure that you have received each of the participant’s signed consent form prior to the focus group session.***

***At the start***

***Welcome participants. Provide participants with a brief recap of the project and plan for the focus group – for example:***

*“Our project is looking at how patient safety recommendations are created by the Health Services Safety Investigations Body (HSSIB), and how these recommendations are received, prioritised and put into action by the receiving organisations.*

***There are no right or wrong answers and there may be differing points of view. Participants should feel free to share their views or experiences even if these differ from what others have said – we do not anticipate consensus, just sharing of experiences and views.***

***Establish ground rules***

- *All participants have a right to their viewpoints and opinions.*
- *All participants have a right to speak without being interrupted.*
- *Please do contribute to the discussion as much as you can, and also allow others time to speak.*
- *We emphasise that what is said in the focus group discussion should remain here. We would advise all participants attending the session to maintain mutual confidentiality towards each other’s participation as far as possible, and particularly towards individual participants’ specific experiences and viewpoints that are expressed here.*

***The focus group discussion will last for about an hour and a half. It will be recorded on Teams but the video will be destroyed once the audio has been extracted. Your data will be anonymised and your responses deidentified.***

***Any questions? Happy to continue?***

- ***Brief round of introductions***
- *Introduce self and colleagues.*
- *Provide very brief introductions about your current work role, in just one or two sentences.*

1. ***Production of HSSIB/HSIB recommendations***

- Tell me a bit about how you produce patient safety recommendations

*[Prompts and probes:*

- *Describe the processes you use for producing recommendations?*
- *Who is involved?*
- *How do the findings of an investigation influence a set of recommendations? Can you give an example?*
- *How are recommendations prioritised?*
- *How do you decide who to target the recommendations at?*
- *How do you know who to direct recommendations to within large organisations?*
- *What knowledge do you draw on to produce them?*
  - *E.g. Use of any established safety science theory and/or evidence-based models, and/or other forms/sources of knowledge?]*

- Tell me your views about the type of patient safety recommendations you produce?

[*Prompts and probes*:

- *What’s more challenging, or easy to develop and why?*
- *To what extent do your recommendations differ to other guidance/recommendations?*
- *What are your views on the language you use in the recommendations (give example)?*
- *And how achievable and actionable do you think they are?*
- *To what extent are there tensions between making recommendations to large organisations and making them actionable?]*
- Is there anything that you think needs to be improved or could be done better/differently in the future with regards to:
- *The content, presentation or format of your recommendations?*
- *Or how you produce your recommendations? – i.e. the processes you use to come up with your recommendations?*
- Why do you produce safety observations as well as recommendations?
- *What are they intended to achieve?*
- *And how are they responded to?*

***2. Reception, implementation, and use of HSSIB’s recommendations***

What happens once you have made a recommendation to an organisation/body,

*[Prompts and probes:*

*How willing and able would the recipients be to act upon the recommendations, do you think?*

*Legally, what are the recipient organisations’ duties once they have received a recommendation?*

*If organisations have to respond to your recommendations – do you grade the responses? If so, how? Criteria?*

*Do you follow up to check what recipient organisations are doing with the recommendations?]*

*Is there a feedback loop from organisations?*

How do recipient organisations act on and implement your recommendations?

*[Prompts and probes:*

*Are there certain types of recommendations/guidance that tend to take priority over other types, or get taken up and implemented more readily?*

*Is there anything about an organisation’s local context that makes it easier or more challenging to act upon and implement received patient safety recommendations or guidance?*

*What resources or support needs to be in place, for organisations to effectively respond to patient safety guidance?*

*Any views or suggestions regarding how recipient organisations may be better able to utilise and act upon your recommendations?]*

How do you know what is tractionable/understand your target audience?

*[Prompts and probes:*

Any stakeholder engagement? When?]

- In your view, what makes a set of recommendations/guidance either easier or more challenging to interpret and implement?

*[Prompts and probes:*

*Can you describe a case where a set of your recommendations, or others, was successfully put into practice? What enabled this?*

- *Can you describe a case where recommendations were not implemented as intended? What were the barriers to implementation?]*

3. ***Overall***

To what extent do you feel that your recommendations make a difference to patient safety?

- What are your views or suggestions regarding whether, and how, your recommendations could be improved in the future?

***Closing***

- We are coming to the end of the focus group now, but before we finish is there anything else we should focus on with regards to how patient safety recommendations are created, received, prioritised and actioned by receiving organisations.
- Do you also have any further comments to add?
- Anything we may have missed?
- Anything that you feel may be important but we have not covered sufficiently today?
- If you have ticked the option on your consent form regarding wanting to receive the findings of this study, then we will send you a summary of the findings once the study has finished and we have completed our analysis.

**Thanks & Close**
