# Supplementary material for: From safety science to operational reality: competing institutional logics in the translation of national patient safety recommendations
Source: Int J Qual Health Care. 2026 Sep 21;38(4):mzag146. doi: 10.1093/intqhc/mzag146 (PMC13690090; doi:10.1093/intqhc/mzag146)
Supplement: Supplementary file 1 [file mzag146_supplementary_data.zip › Supplementary material 3.docx]

# Supplementary material 3

List of organisations

These organisations included NHS England, the Care Quality Commission (CQC), the National Institute for Health and Care Excellence, the National Institute for Health and Care Research, the General Medical Council, the Royal College of Emergency Medicine, the Royal College of Physicians, and the Royal College of Anaesthetists
