# Supplementary material for: From safety science to operational reality: competing institutional logics in the translation of national patient safety recommendations
Source: Int J Qual Health Care. 2026 Sep 21;38(4):mzag146. doi: 10.1093/intqhc/mzag146 (PMC13690090; doi:10.1093/intqhc/mzag146)
Supplement: Supplementary file 1 [file mzag146_supplementary_data.zip › Supplementary material 4 Topic Guide for interviews 11.9.2024.docx]

**From Recommendation to Action (ReACTION): Exploring the Translation of National Guidance into Organisational Change for Patient Safety**

**Interview Topic Guide**

****Note to interviewer:***

***Remember to provide the participant with a brief recap of the project and plan for the interview – for example:***

*“Our project is looking at how patient safety recommendations are created by the Health Services Safety Investigations Body (HSSIB), and how these recommendations are received, prioritised and put into action by the receiving organisations. We will start the interview with a brief introduction about your current work role and/or expertise, then talk about your experiences and/or views relating to the production of patient safety recommendations, and then move on to talk about how patient safety recommendations are received, interpreted, prioritised and put into action by organisations who receive the recommendations.”*

***Highlight that the interview will be recorded, outlining briefly how the recording will be managed, stored and used. Also highlight that the interview data will be anonymised and their responses deidentified.***

***Check the participant is happy with the above prior to commencing, and that they have signed the consent form. Include prompt that participant can pause or stop the interview at any time.***

***Participant background***

- Can you tell me about your work-role and how it relates to patient safety?

***Production of HSSIB/HSIB recommendations***

- Can you tell me what you know about how HSSIB/HSIB produces its patient safety recommendations?
- What do you think about the processes HSSIB/HSIB uses for coming up with its patient safety recommendations?
  - *Do you feel you always know how HSSIB came about with a recommendation and the evidence behind it?*
- Which investigation(s) have you been involved in? and how involved have you been in the production of these r?
- To what extent was your involvement at the right point?
- What is your opinion of the recommendations produced by HSSIB/HSIB?

***Reception, implementation, and use of HSSIB’s recommendations***

Can you tell me about your experience of acting on and implementing HSSIB/HSIB’s recommendations?

What mandate do you have to act on the recommendations from HSSIB?

What effect do you feel the recommendations have on patient safety?

*Are they specific enough to improve patient safety?*

What happens once you have accepted a recommendation from HSSIB, as an organisation?

- In what ways does your organisation’s local context help or hinder, how you act upon and implement received patient safety recommendations or guidance?

Can you tell me how your organisation navigates and responds to patient safety recommendations received from various sources?

- Are there certain types of recommendations/guidance that tend to take priority over other types, or get taken up and implemented more readily?
- *Why do you think this is the case?*
- *To what extent do regulatory pressures influence the uptake of recommendations?*
- *and public/media scrutiny?*

What factors, if any, make recommendations easier/harder to implement?

To what extent does the status of HSSIB

affect how the recommendations are received compared to other recommendations you may receive?

What proportion of recommendations you receive are new compared to what is already in the pipeline?

How helpful is it to receive reinforcement of recommendations?

Do you have any further suggestions regarding how HSSIB/HSIB’s recommendations could be improved for the future?

What resources exist, or need to be in place, for organisations to more effectively respond to patient safety guidance?

How can you make it easier for HSSIB to find the right person to address the recommendation?

***Close of interview:***

Do you also have any further comments to add at this point regarding anything we may have missed in our discussion, or raise anything that you feel may be important but I may not have asked you about?

- If any clarifications are needed, may we contact you via email?
- If you have ticked the option on your consent form regarding wanting to receive the findings of this study, then we will send you a summary of the findings once the study has finished.

**Thanks & close of interview**
