# Supplementary material for: From safety science to operational reality: competing institutional logics in the translation of national patient safety recommendations
Source: Int J Qual Health Care. 2026 Sep 21;38(4):mzag146. doi: 10.1093/intqhc/mzag146 (PMC13690090; doi:10.1093/intqhc/mzag146)
Supplement: Supplementary file 1 [file mzag146_supplementary_data.zip › Supplementary material 5 Focus group coding - 12-05-2026.docx]

ReACTION Focus group RECS

Codes

| Name | Description |
| --- | --- |
| A human industry not just technical |  |
| challenge of multiple stakeholders |  |
| Changes over time |  |
| Collaboration |  |
| Consultation process |  |
| Competition |  |
| Develop from evidence |  |
| Dumbing down |  |
| Easy or hard recs |  |
| National recs trickle down |  |
| effectiveness of recs |  |
| Filling a gap |  |
| HSSIB as orchestrator |  |
| identifying the right stakeholders |  |
| Personal experience |  |
| Improving recs |  |
| Independence |  |
| lack of understanding re safety and safety science |  |
| Making the situation safer |  |
| Not a regulator |  |
| patient safety concerns process and response |  |
| Response to recs - process |  |
| Accountability and ownership of risk |  |
| follow up |  |
| grading |  |
| Impact |  |
| Monitoring recs |  |
| Reception to recs |  |
| Role of leadership |  |
| safety science logic |  |
| Stakeholder involvement |  |
| Suggestions not solutions |  |
| tension between collaboration and independence |  |
| transparency |  |
| what makes their recs different |  |
