# Supplementary material for: From safety science to operational reality: competing institutional logics in the translation of national patient safety recommendations
Source: Int J Qual Health Care. 2026 Sep 21;38(4):mzag146. doi: 10.1093/intqhc/mzag146 (PMC13690090; doi:10.1093/intqhc/mzag146)
Supplement: Supplementary file 1 [file mzag146_supplementary_data.zip › Supplementary material 6 interview coding - 13-03-2026.docx]

ReACTION recipients interviews

Codes

| Name | Description | Files | References |
| --- | --- | --- | --- |
| background and role |  | 7 | 8 |
| context of organisation |  | 6 | 7 |
| examples of disagreements |  | 3 | 9 |
| general opinion of HSSIB recs |  | 8 | 13 |
| HSSIB status |  | 8 | 21 |
| impact on patient safety |  | 7 | 11 |
| specific enough |  | 5 | 9 |
| Involvement |  | 0 | 0 |
| How involved |  | 7 | 11 |
| right point |  | 7 | 11 |
| which investigations |  | 7 | 8 |
| Navigating recs |  | 4 | 9 |
| other |  | 3 | 8 |
| Prioritising recs |  | 7 | 19 |
| process of producing recs |  | 8 | 12 |
| process of receiving acting on recs |  | 8 | 24 |
| mandate |  | 5 | 6 |
| repetition |  | 8 | 13 |
| resources needed |  | 6 | 6 |
| responsibility and accountability |  | 1 | 2 |
| right person to contact |  | 7 | 8 |
| suggestions to improve |  | 7 | 16 |
| tensions |  | 3 | 4 |
| what makes harder to implement |  | 7 | 14 |
| what makes it easier to implement |  | 8 | 17 |
